# The optimal shape of elastomer mushroom-like fibers for high and robust adhesion

**DOI:** 10.3762/bjnano.5.74

**Published:** 2014-05-14

**Authors:** Burak Aksak, Korhan Sahin, Metin Sitti

**Affiliations:** 1 Department of Mechanical Engineering, Texas Tech University, Lubbock, TX 79409, USA; 2 Aerospace Engineering, University of Illinois at Urbana-Champaign, Urbana, IL 61801, USA; 3 Department of Mechanical Engineering, Carnegie Mellon University, Pittsburgh, PA 15213, USA

**Keywords:** gecko, mushroom-like fibers, adhesion

## Abstract

Over the last decade, significant effort has been put into mimicking the ability of the gecko lizard to strongly and reversibly cling to surfaces, by using synthetic structures. Among these structures, mushroom-like elastomer fiber arrays have demonstrated promising performance on smooth surfaces matching the adhesive strengths obtained with the natural gecko foot-pads. It is possible to improve the already impressive adhesive performance of mushroom-like fibers provided that the underlying adhesion mechanism is understood. Here, the adhesion mechanism of bio-inspired mushroom-like fibers is investigated by implementing the Dugdale–Barenblatt cohesive zone model into finite elements simulations. It is found that the magnitude of pull-off stress depends on the edge angle θ and the ratio of the tip radius to the stalk radius β of the mushroom-like fiber. Pull-off stress is also found to depend on a dimensionless parameter χ, the ratio of the fiber radius to a length-scale related to the dominance of adhesive stress. As an estimate, the optimal parameters are found to be β = 1.1 and θ = 45°. Further, the location of crack initiation is found to depend on χ for given β and θ. An analytical model for pull-off stress, which depends on the location of crack initiation as well as on θ and β*,* is proposed and found to agree with the simulation results. Results obtained in this work provide a geometrical guideline for designing robust bio-inspired dry fibrillar adhesives.

## Introduction

We need to look no further than nature to find inspiration for many of the technologies we work on today. One such field that observations on natural systems have impacted significantly in the recent years is adhesive technologies. While conventional adhesives rely on very soft materials or viscous liquids, nature offers a unique system composed of adhesive elements made of relatively rigid materials. These adhesive elements are comprised of millions of tiny fibers varying in size and geometrical complexity depending on the animal that bears them [1]. Some insects, spiders, and anoles have fibers with effective diameters of the order of micrometers. Other animals such as the gecko lizard bear micro-scale stalks, which branch down to nano-scale fibers forming intricate hierarchical structures. The common aspect of fibrillar structuring is its ability to conform to the adhering surface, improve contact area and create an attractive force between individual fibers and the surface. In geckos, this attractive force is believed to arise from van der Waals interactions between the terminal end of an individual fiber and the surface [2–3]. A recent study by Hsu et al. [4] suggests that the presence of phospholipids on the tips of the fibers aid in adhesion. It has also been shown that humidity levels change the clinging ability of geckos significantly [5–8]. Regardless of the adhesion mechanism, the cumulative effect from the adhesion contribution of every fiber in contact is capable of generating adhesive strengths up to 100 kPa [9] as observed for the gecko lizard. A great deal of research has been performed to analyze the structure of natural fibrillar adhesives and measure their performance [2–3,6,8–11], to understand the main principles of enhanced adhesion [12–25], and to fabricate synthetic counterparts of biological fiber adhesives [16,21,26–36].

**Figure 1:**
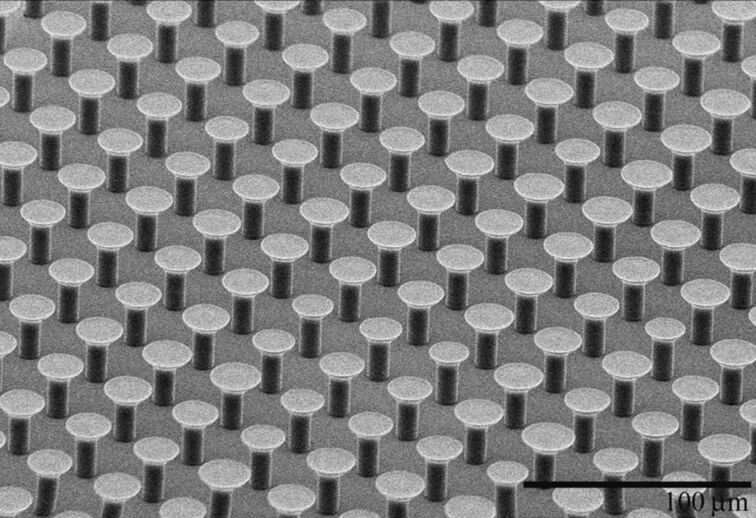
Scanning electron microscope image of polyurethane mushroom-like fibers with 4 µm stalk radius, 8 µm tip radius, and 20 µm height.

A common aspect of natural fibers among species, which is of interest in this work, is that the cross section of a natural fiber is rarely constant along its longitudinal axis. It increases close to its terminal end forming what is referred to in literature as *mushroom/spatulae-shaped fibers* [2,21,30,33,37]. While initial fabrication attempts for synthetic adhesives were limited to constant cross section cylindrical fibers [16,20,34–35], the realization of the actual shape of natural fibers has led to synthetic mushroom-like fibers (Figure 1). Adhesives comprised of mushroom-like fibers have shown significant improvements over cylindrical fibers. Furthermore, measured adhesive strengths have matched, and in some instances such as smooth surface applications, surpassed the adhesive strengths recorded for gecko footpads [21,30,33,37].

Work by del Campo et al. [21] reports enhancements in pull-off loads as much as 40-fold with mushroom-like fibers over cylindrical fibers of equal height and stalk radius. Interestingly, for the mushroom-shaped fibers that exhibited this enhancement, the contact area is only 1.7 times the contact area of flat tip cylindrical fibers. This fact points to the existence of an adhesion enhancement mechanism other than just the increase in contact area with mushroom-like fibers. Spuskanyuk et al. [38] used the assumption of pre-existing annular cracks and Griffith’s energy criterion for crack propagation to study the enhancement mechanism for mushroom-like fibers. They concluded that for a given load and crack length, the relatively higher energy release rate for a cylindrical fiber induced pull-off at significantly lower loads than mushroom-like fibers. In their analysis, the ratio of pull-off force of a mushroom-like fiber to that of a cylindrical fiber varies significantly depending on the size of the annular crack. Carbone et al. [39] also looked at the adhesion mechanism of mushroom-like fibers and concluded that these fibers are superior to cylindrical fibers in their ability to eliminate stress singularities as well as stabilize defects at the interface. They considered edge angles to be 90° and theoretically determined pull-off stress as well as propose maps for detachment behavior of these structures. Carbone and Pierro [40] performed further optimization studies to determine an optimal shape for mushroom-like fibers based on the microfiber geometry fabricated by Gorb et al. [33]. We compare their results with findings in this work in section Results in detail.

In this work we study the effect of geometry, defined by the edge angle θ and the ratio of the tip radius to the stalk radius β*,* on pull-off stress of mushroom-like fibers by using a cohesive zone model and finite elements (FE) simulations. Description of the cohesive zone model and numerical simulations are included in sections “Cohesive zone model” and “Numerical simulations”, respectively. After that, the results of the finite element simulations are presented, and in the subsequent section the detachment behavior of individual fibers, the effect of tip apex shape and friction, a model to estimate pull-off stress for mushroom-like fibers, and a comparison between cylindrical and mushroom-like fibers in terms of pull-off stress are discussed.

## Cohesive zone model

Adhesion problems can be studied by using a cohesive zone model such as the Dugdale–Barenblatt (DB) model [41–42]. It is a simple cohesive zone model in which the interface separates when the normal interfacial stress reaches the theoretical strength of the interface σ_o_. The interface continues to separate at σ_o_ until the separation reaches a critical distance δ_c_, after which the interface can no longer support stress, resulting in a crack to initiate. The region where the separation of interface occurs is referred to as the *cohesive zone*. In this model, the work of adhesion is given by w_adh_ = σ_o_δ_c_. Tang et al. [15] found the pull-off force of a soft, elastic cylindrical fiber in contact with a rigid flat surface whose height is much larger than its radius by using the DB cohesive zone model. According to their study, normalized pull-off stress Φ ≡ σ_s_/σ_o_ depends on a single dimensionless parameter χ defined as

[1]
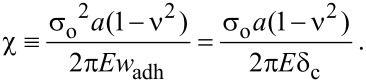


Here, *a* is the radius, *E* is the elastic modulus, and ν is the Poisson’s ratio of the fiber. The dimensionless parameter χ is the ratio of the fiber radius to a length-scale related to the dominance of the interfacial adhesive forces. Hence, when *χ* << 1, an attractive stress that is equal to the intrinsic adhesive stress covers the entire fiber tip and the pull-off stress approaches the theoretical limit, Φ = 1 (i.e., σ_s_ = σ_o_). This regime is referred to as the *flaw-insensitive regime*. On the other hand, when χ >> 1, σ_o_ acts over a small portion of the interface, which results in the pull-off stress being much smaller than the theoretical limit, σ_s_ << σ_o_. This regime is referred to as the *flaw-sensitive regime*. The non-dimensional parameter χ is also relevant to the adhesion problem of mushroom-like fibers. Thus, pull-off results will be presented as a function of χ. Later in section Discussion, a pull-off stress model based on χ will be presented.

## Numerical simulations

**Figure 2:**
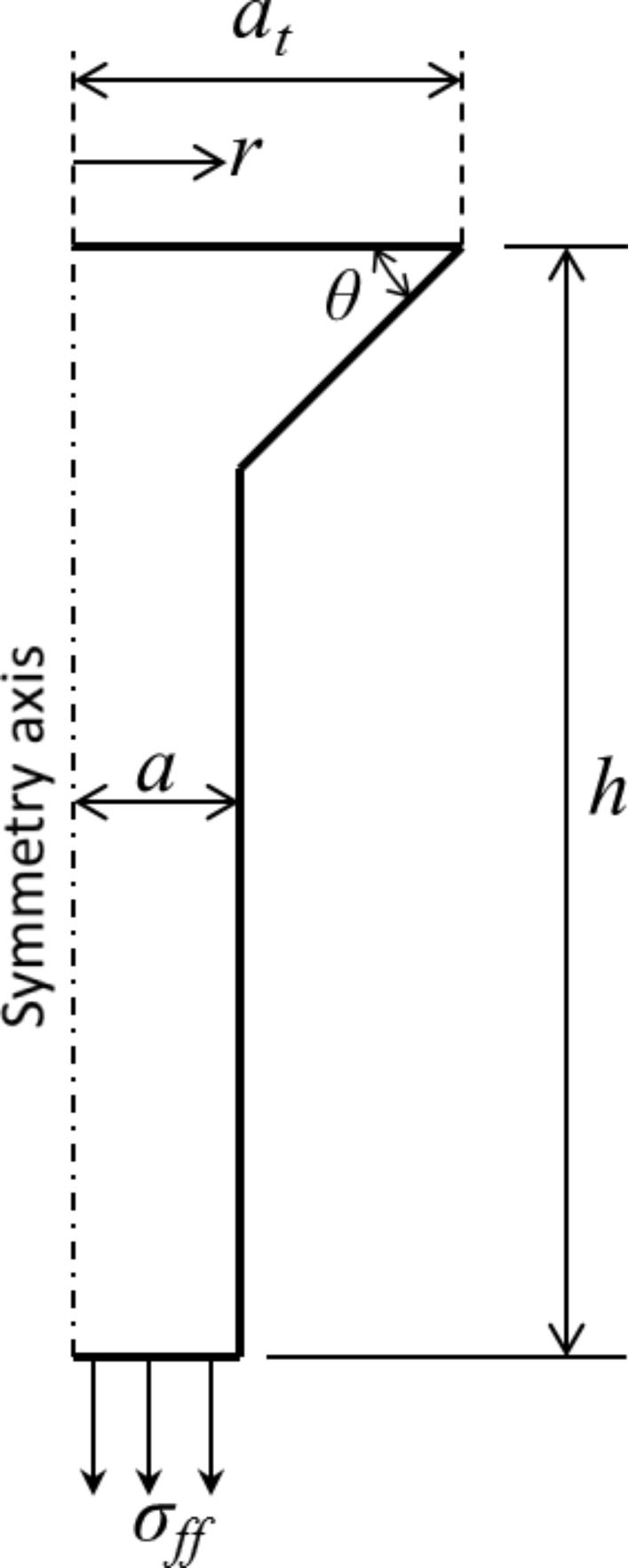
Two-dimensional axial symmetry model for a mushroom-like fiber. The tip (top surface) is fixed in radial direction to simulate full-friction contact. DB cohesive zone model is implemented at the tip of the fiber in FE simulations.

**Figure 3:**
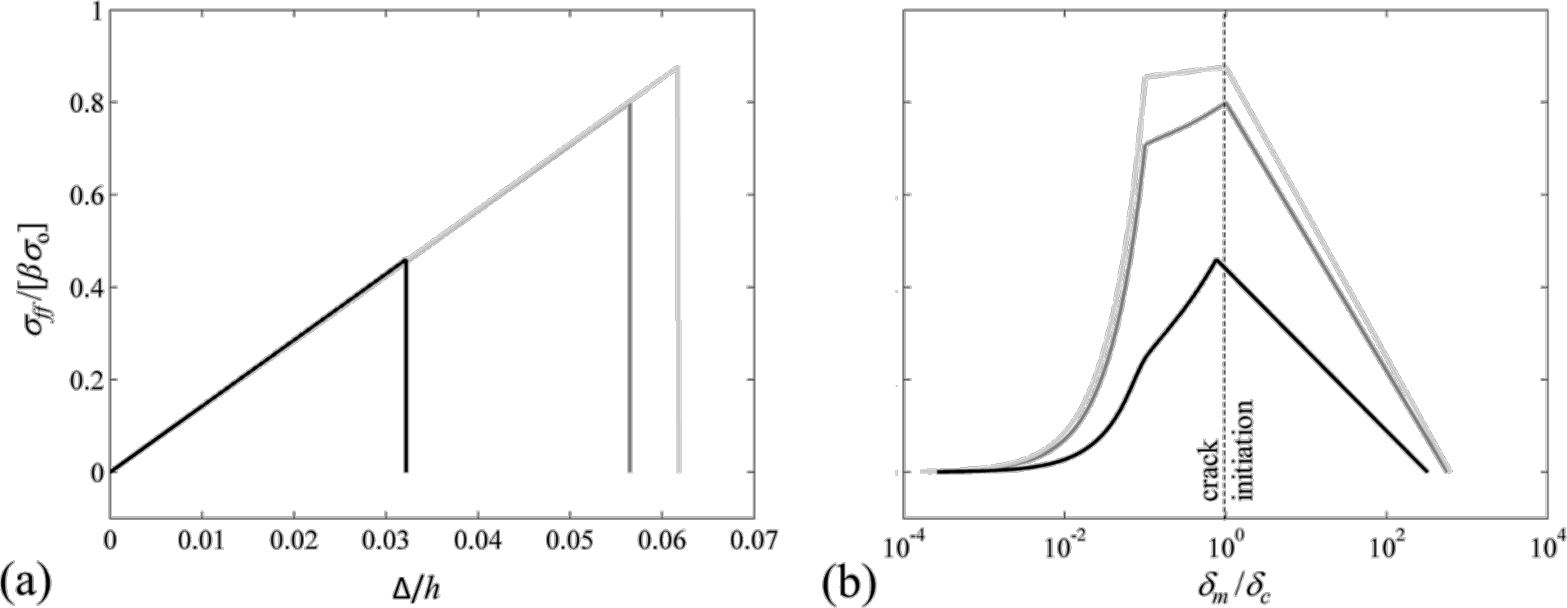
Average tensile stress at the tip of the fiber (a) as a function of normalized far field displacement Δ/*h*, and (b) as a function of normalized maximum interfacial separation δ_m_/δ_c_ for β = 1.2 and θ = 25° (dark gray), θ = 45° (light gray), and θ = 75° (black). Here, δ_c_ = 1 nm (χ = 6). Peaks in each plot for specific θ coincide and correspond to normalized pull-off stress Φ. Tensile load drops immediately after the maximum interfacial separation reaches the critical separation indicating that the contact is unstable following crack initiation. The discontinuity at δ_m_/δ_c_ ≈ 0.1 prior to crack initiation in (b) marks the instant when a cohesive zone starts to form.

Simulations are performed for a mushroom-like fiber illustrated in Figure 2 by using the analysis software COMSOL MultiPhysics 4.3 FE. It is assumed that the fiber is in full friction contact with a rigid smooth surface, which is in line with our observations during experiments with mushroom-like polyurethane fibers [32,37]. The DB cohesive zone model is implemented and modified slightly to avoid divergence of the numerical solution. Step function from zero stress to σ_o_ in the cohesive zone model is replaced with a high stiffness relation between the attractive stress and interfacial opening where the interface is required to separate by 10% of δ_c_ before cohesive zone forms (i.e., σ_o_ is reached) [24]. Unless stated otherwise, the simulation parameters are *a* = 1 µm, *E* = 3 MPa, ν = 0.5 and σ_o_ = 100 kPa. The tip radius *a*_t_ is varied from 1.05 µm to 2 µm while the edge angle θ is varied from 25 to 80°. It is important to note that the tip corner (wedge) is chosen so that the wedge angle is also equal to θ*.* The effect of the wedge angle being different from θ is briefly addressed in the section Discussion. The height of the fiber *h* is fixed at 10 µm. Dimensionless parameter χ is varied by changing δ_c_ for fixed *a*_t_, *E*, ν and σ_o_. While applying a displacement Δ gradually to the base of the fiber, the pull-off load is determined from the far field tensile stress σ_ff_ the fiber bears when the maximum interfacial separation equals δ_c_. We found that the tensile load reaches its maximum at the instant δ_c_ is reached at the interface (see Figure 3). Knowing the pull-off load *p*_s_, the pull-off stress is calculated from σ_s_ = p_s_/(π*a*_t_^2^).

## Results

**Figure 4:**
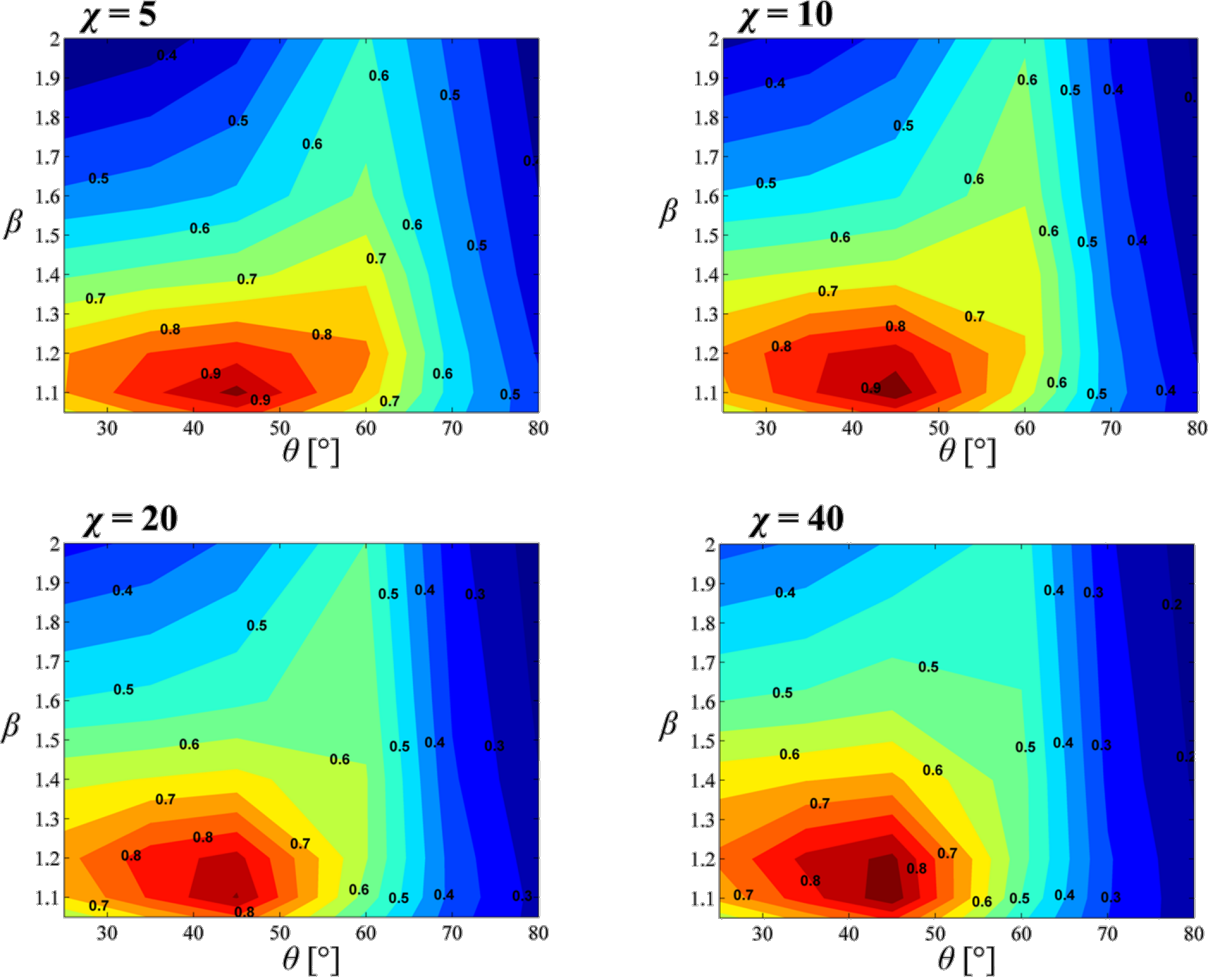
Normalized pull-off stress Φ contour plots for χ = 5 (top left), χ = 10 (top right), χ = 20 (bottom left), and χ = 40 (bottom right) as a function of β and θ. The peak Φ for each case lies within β = 1.1–1.2 and θ = 45°.

**Figure 5:**
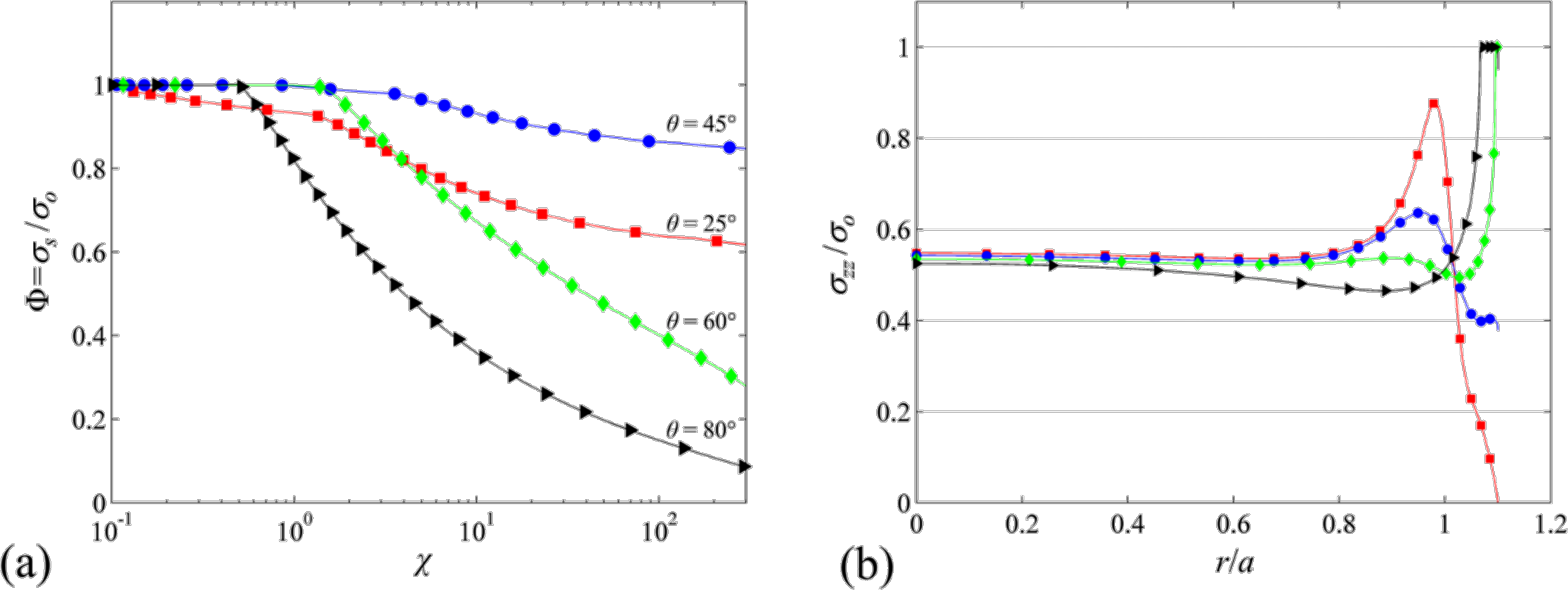
(a) Normalized pull-off stress as a function of χ for β = 1.1 and θ = {25°, 45°, 60°, 80°}. (b) Normalized stress at the tip for Δ/*h* = 0.0217 for the same β and θ as in (a). A cohesive zone is present at the edge both for θ = 60° and θ = 80°, while it has not formed for θ = 25° and θ = 45° yet.

Pull-off stress for all tip-to-base ratios β ≡ *a*_t_ / *a* and edge angles θ are shown for select χ values in Figure 4. Contour plots show clear peaks at β = 1.1–1.2 and θ = 45° suggesting that these values are optimal for maximum pull-off stress. As expected, the peak pull-off stress drops with increasing χ with the highest value at Φ = 0.97 for χ = 5 and the lowest value at Φ = 0.88 for χ = 40 both obtained for β = 1.1 and θ = 45°. Figure 5a shows pull-off stress for β =1.1 for all θ as a function of χ. In line with the data presented in Figure 4, θ = 45° yields the highest pull-off stress for all χ, except when χ << 1. In the regime where χ << 1, pull-off is flaw-insensitive and it is expected that Φ = 1 regardless of β*,* θ*,* and χ. This confirms findings by Tang et al. [15] and Gao and Yao [12].

In Figure 5a, one observes that for θ ≤ 45°, the pull-off stress saturates towards a constant value as χ increases. In contrast for θ > 45°, the pull-off stress continuously drops with increasing χ. The dependence of pull-off stress on the edge angle at the limit χ → ∞ (i.e., δ → 0) can be explained by using the study of Bogy [43] on stress singularities at bimaterial wedge interfaces. For a soft incompressible elastomer (i.e, Poisson’s ratio ν = 0.5) in full-friction contact with a rigid substrate, stress at the edge of the fiber tip is finite for θ ≤ 45° and singular for θ > 45°, (see Supporting Information File 1 for details). Stress profiles of a mushroom-like fiber with β = 1.1 and θ = {25°,45°, 60°, 80°} are shown for a far field displacement of Δ/*h* = 0.0217 in Figure 5b. Due to the singularity, a cohesive zone is present at the edge of the fiber both for θ = 60° and θ = 80° while cohesive zone has not formed for θ = 25° and θ = 45° yet. Let us assume that δ_c_ = 0, which implies that pull-off will occur shortly after the maximum tensile stress at the interface reaches σ_o_. If there is a stress singularity at the edge of the tip, normal stress will be equal to σ_o_ at the edge the moment a tensile load is applied to the fiber. The interface will open starting at the edge and pull-off will depend on whether this opening is less than or equal to δ_c_. On the other hand, if stress is finite everywhere at the interface, a sufficiently large tensile load has to be applied before a cohesive zone starts forming. This implies that regardless of the value of δ_c_, pull-off load has a finite lower limit if θ ≤ 45°. The existence of this lower limit provides robust adhesion because regardless of the value of χ, one can expect to obtain a pull-off stress equal to this lower limit in the least. In particular for fibers with θ = 45° and β = 1.1, this lower limit for normalized pull-off stress is remarkably Φ = 0.85 which ensures high and robust adhesion.

Let us compare our results with previous findings of Carbone and Pierro [40]. Carbone and Pierro used the Griffith method to design an optimal fiber shape based on the synthetic adhesives developed by Gorb et al. [33]. They concluded that for optimal adhesion, which is essentially a measure of pull-off force for a single fiber, 2 ≤ β ≤ 3 and *s*/*a* = 0.2–0.3. Here, *s* is the thickness of the circular disk at the terminal end and *s* would be zero for our case. The discrepancy between their findings and ours can be attributed to three main factors. In their analysis, the apex angle is fixed at 90°, which leads to a singularity at the edge for fiber geometries if β < 2. As expected, the tip-size should be large to eliminate this singularity (i.e., equivalent stress intensity factor at the contact edge approaches zero), which necessitates the condition for 2 ≤ β ≤ 3. They then adjust *s* to avoid stress peaks and ensure uniform stress distribution at the tip and find that *s*/*a* =0.2–0.3 yields a peak free stress distribution. In our case, the apex angle is a variable and for θ ≤ 45°, the singularity at the edge disappears regardless of the value of β. Therefore, the condition β > 2 is not a necessary condition for the fiber geometry considered in this work, which is based on the fiber shapes included in Figure 1 and previous studies [32,37]. We find β = 1.1–1.2 to be optimal for uniform stress distribution. The second factor is the difference in what is considered optimal. In their study, Carbone and Pierro considered the far field stress, a measure for the pull-off stress of a single fiber, as the optimization function. Thus, the optimal parameters offered are to maximize single fiber pull-off load. They include a qualitative argument that increasing the tip size reduces the maximum number of fibers per unit area, and therefore β > 3 should be avoided. In our study, the optimization function is the pull-off stress per unit contact area of a single fiber, which quantitatively factors in the effect of contact area. The third factor is that they determine their equivalent stress intensity factor using both mode I and mode II stress intensity factors. Mode II (fracture mode) opening was not considered in our study and could be important especially for highly adhesive interfaces. We therefore expect discrepancies between the optimal solutions due to the differences in the model geometry, optimization function, and adhesion modeling.

## Discussion

### Crack initiation and pull-off model

The location of crack initiation (i.e., where the interface opening equals δ_c_) depends both on tip shape parameters θ and β, and the value of χ. For θ ≤ 45°, since normal stress is finite everywhere at the tip, cohesive zone forms when and where the maximum normal stress reaches σ_o_. This location corresponds to *r*/*a* = 0 (center) for θ = 45° and all β (refer to Figure 5b for normal stress profiles). Simulation results show that the location of crack initiation is independent of χ for this set of tip parameters. For θ > 45° on the other hand, the crack initiation is always at the edge for sufficiently large χ. For certain combinations of β and θ, for instance β = 1.4 and θ = 60°, although the normal stress at the tip is highest at the edge, it does not increase monotonically from the center to the edge of the tip. Stress profile has a minimum at *r*/*a* ≈ 1 (Figure 5b). For sufficiently low χ, gradually increasing far field displacement causes a cohesive zone to form at the edge first. Since δ_c_ is relatively large for low χ, increase in tension does not immediately result in a crack to initiate at the edge. In the meantime the stress in the center gradually increases reaching σ_o_ at which a second cohesive zone starts to form. The center separates faster than the edge, which results in a crack to initiate at the center. It is observed that crack initiation switches to the center of the fiber if stress at the center is able to reach σ_o_ prior to a crack initiating at the edge.

Similar to the pull-off stress model proposed by Tang et al. [15], derived with the assumption that the size of the cohesive zone is much smaller compared to the tip radius, pull-off stress can be estimated according to where the crack initiates as

[2]
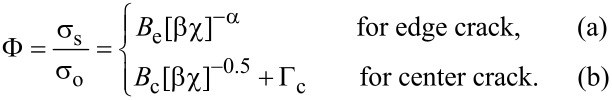


**Figure 6:**
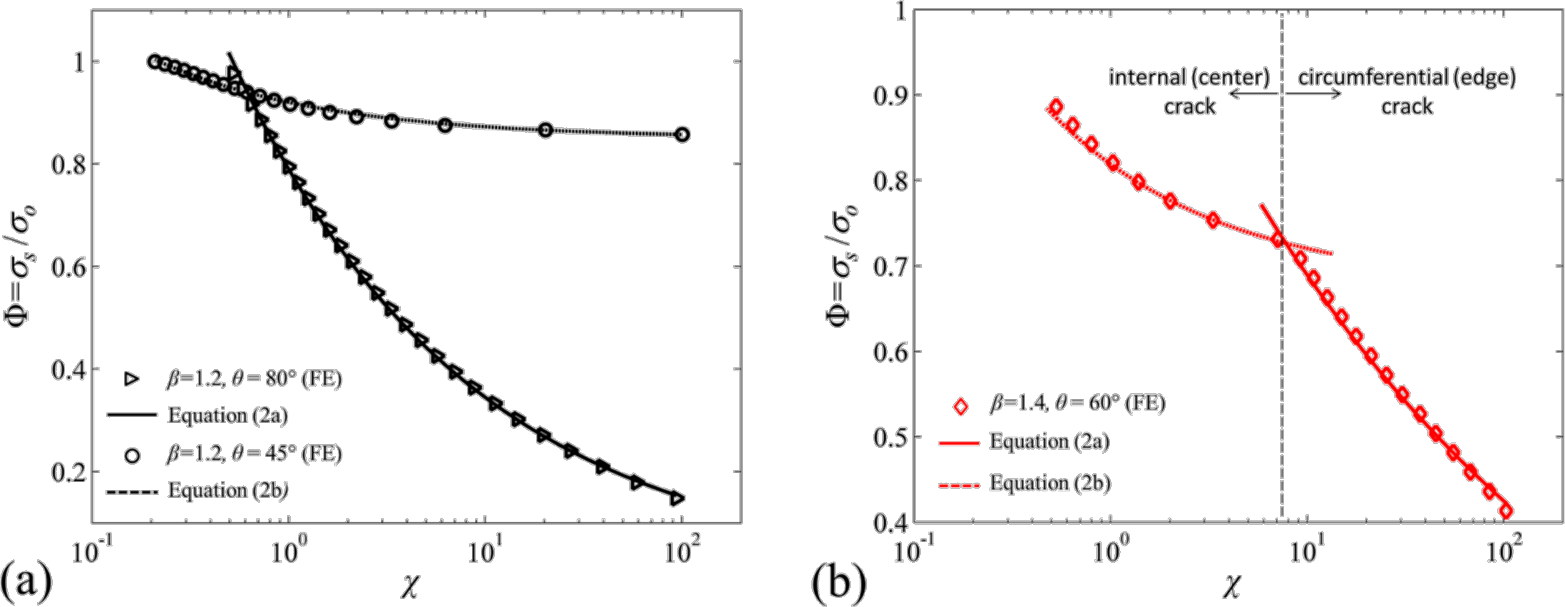
(a) Simulation results (triangle markers) for β = 1.2, θ = 80° for which a crack initiates at the edge for all χ. Solid line represents Equation 2a with *B*_e_ = 0.84 and α = 0.36. Also included are the simulation results (circle markers) for β = 1.2 and θ = 45° for which a crack initiates at the center for all χ. Dashed line represents Equation 2b with *B*_c_ = 0.076 and Γ_c_ = 0.85. (b) Simulation results for β = 1.4, θ = 60° for which a crack initiates at the edge for χ > 7 and at the center for χ < 7. Solid line represents Equation 2a with *B*_e_ = 1.2 and α = 0.21. Dashed line represents Equation 2b with *B*_c_ = 0.17 and Γ_c_ = 0.68.

Here, α can be found for a given edge angle *θ* using Equation S1 in Supporting Information File 1. For a crack initiating at the center, there is a square-root singularity and thus α = 0.5 as shown in Equation 2b. The numerical constants *B*_e_ and *B*_c_ are form factors that are determined by fitting Equation 2a and Equation 2b to the simulation data for given β, θ, and χ. The constant Γ_c_ is approximately the value of the pull-off stress when the crack initiation switches from the edge to the center of the fiber for θ > 45°. It is determined by fitting Equation 2b to simulation data. For θ ≤ 45° and a crack initiating at the center for all χ, Γ_c_ is the lower bound for normalized pull-off stress at the limit χ → ∞. Figure 6 shows the simulation data and the model fits using Equation 2a and Equation 2b.The proposed model is in agreement with the simulation data except when χ → 0. Equation 2 is not valid in this regime as the cohesive zone occupies a relatively larger portion of the tip. Additionally, for certain combinations of β and θ, a crack initiates at *r*/*a* ≈ 1 due to a stress peak at this location. Equation 2 is not valid in this case.

Crack initiation at the center of the fiber shown in simulations was also seen experimentally by Varenberg et al. [44], Heepe et al. [45], and Murphy et al. [32]. Both Heepe et al. and Varenberg et al. presented experimental results with mushroom-like fibers by using high speed imaging and showed that the detachment of the fiber was initiated with an internal crack. Similarly, Murphy et al. observed under tensile loading of polyurethane mushroom-like fibers that an internal crack formed and propagated in a matter of milliseconds leading to contact failure.

All the simulation results show that Φ = 1 in the flaw-insensitive regime (χ << 1) regardless of the value of θ and β. This is in agreement with Gao and Yao [12], and Tang et al. [15], who suggest that for fibers with small radii (i.e., small χ for a given material and adhering surface), pull-off stress is equal to the intrinsic adhesive stress and is independent of the tip shape.

### The effect of interfacial friction

The choice of fiber material and the adhering surface could result in friction ranging from no friction to full friction between the fiber tip and the adhering surface. As shown in Supporting Information File 1 (Figure S1), the magnitude of singular stress at the vicinity of the tip apex is higher for full friction interfaces than frictionless interfaces. Additionally, the limit wedge angle for finite stress increases from 45 to 90°. Thus, the full friction case represents the worst case scenario in terms of the stress singularity at the apex of the mushroom-like fiber and the optimal parameters found in this study, namely β = 1.2 and θ = 45°, would be the conservative choices for fibrillar adhesive design. Note that Equation 2a and Equation 2b are still applicable for frictionless contact where one can obtain α by using Equation S2 rather than Equation S1 in Supporting Information File 1.

### Pull-off stress comparison between mushroom-like and cylindrical fibers

For χ << 1, i.e., for very small fiber radii (typically less than 1 µm), the pull-off stress equals intrinsic adhesive stress and is insensitive to the tip shape. This is in agreement with our results. However, for large χ, the tip shape significantly affects adhesion. Del Campo et al. [21] measured pull-off loads for mushroom-like fibers and cylindrical fibers of the same height, stalk radius and packing density. While they measured approximately 0.7 mN for cylindrical fibers with a hemispherical glass indenter, the measured pull-off load was approximately 28 mN with mushroom-like fibers, a 40-fold increase. Reported values are approximate pull-off loads near saturation as interpreted from the graphical data presented in [21]. Let us define an enhancement factor *e* as the ratio of the pull-off load between two different fiber arrays. For an experiment that uses hemispherical glass indenter with a radius much larger than the dimensions of an individual fiber in the array, the enhancement factor of mushroom-like fibers over the cylindrical fibers with the same packing density, stalk radius, height, and material becomes

[3]
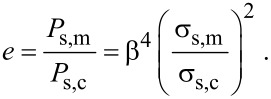


Here, *P*_m_ and *P*_c_ are the pull-off loads for the mushroom-like and cylindrical fiber arrays, respectively. The derivation of Equation 3 is detailed in Supporting Information File 1. Their experiments were carried out with polydimethylsiloxane (PDMS) cylindrical and mushroom-like fibers. For cylindrical fibers, *a* = 10 µm and *h* = 25 µm. The tip radius for mushroom–like fibers were *a*_t_ = 13 µm and they shared the same *a* and *h* with the cylindrical fibers. For both samples, the packing densities (number of fibers per unit area) were the same. According to our model, β = 1.3 and although the edge angle was not reported in their work, we will assume θ = 45° for simplicity. For β = 1.3 and θ = 45°, a conservative estimate of normalized pull-off stress according to our model is σ_s,m_ = Γ_c_σ_o_ = 0.74σ_o_. For cylindrical fibers, σ_s,c_ = 0.83χ^−0.4^σ_o_ as shown by Tang et al. [15]_._ For the described fiber arrays, Equation 3 becomes *e* = 2.27χ^0.8^. Let us assume PDMS to be incompressible (ν = 0.5) with elastic modulus *E* = 1.42 MPa [16]. For glass–PDMS contact, *w*_adh_ = 25 mJ/m^2^ [46]. Let us also assume an intrinsic adhesive stress of *σ*_o_ = 1 MPa, a reasonable value for PDMS [47–49]. By inserting the materials properties, interfacial properties and fiber dimensions into Equation 1, one finds χ = 33.6 and in turn *e* = 38. This estimate is close to *e* = 40 that del Campo et al. [21] obtained in their measurements.

### The implications tip apex shape on pull-off stress

**Figure 7:**
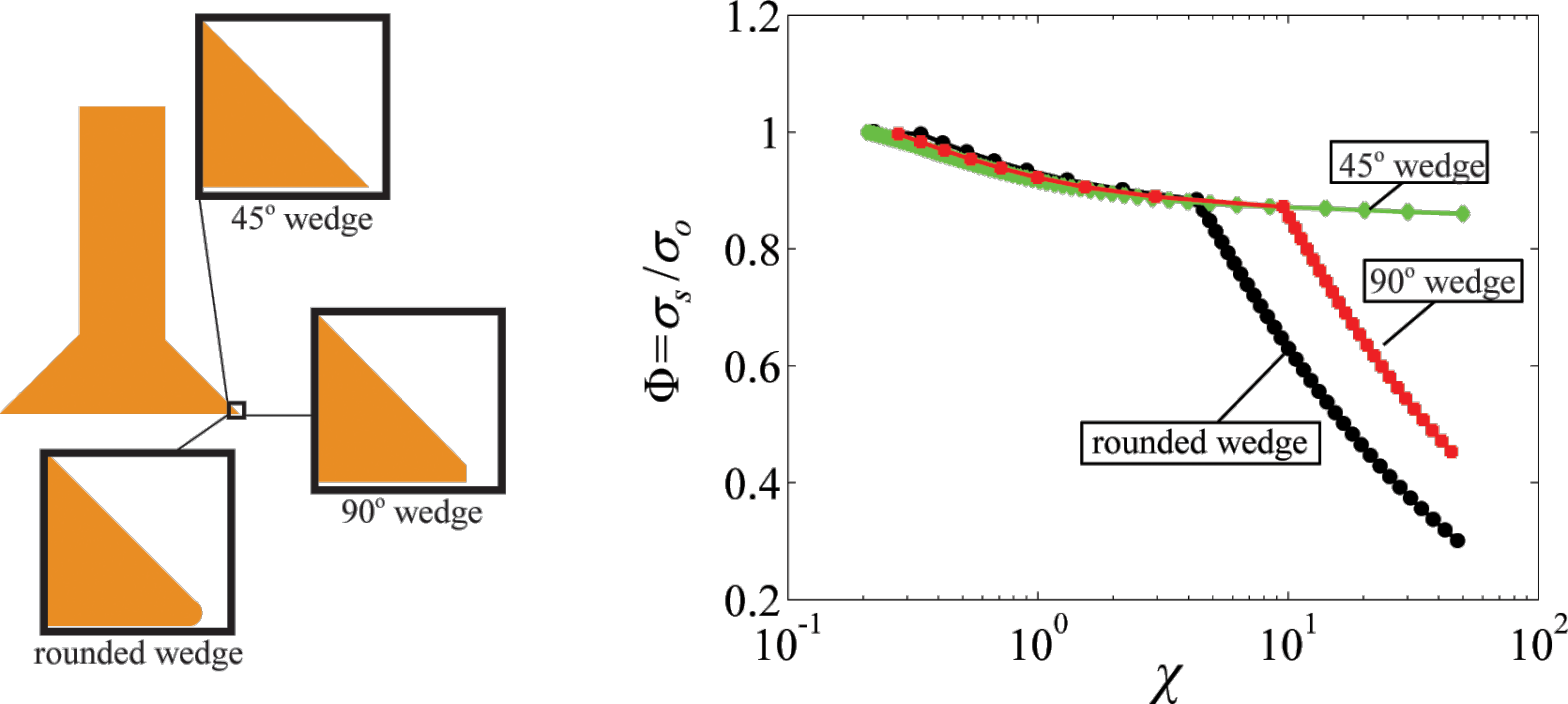
(left) Illustration of three different wedge angles for the mushroom-tipped fibers with β = 1.2 and θ = 45°. (right) Simulation results for 45° wedge (diamond markers), 90° wedge (square markers), and rounded wedge (circular markers).

The manufacturing technique used to fabricate mushroom-like fibers may not yield a sharp corner (i.e., wedge) for individual fibers at the edge of the tip [21,30,32,37,44]. This implies that the wedge angle will be different from the edge angle defined in this work, which may significantly affect the pull-off stress. To demonstrate this effect, we performed simulations for mushroom-like fibers with β = 1.2 and θ = 45° employing three different wedge shapes; namely 45° wedge, 90° wedge and rounded wedge. The radius of curvature was set to 10 nm for the rounded wedge. In case of the 90° wedge, a 10 nm high rectangle was added to the tip (see Figure 7). For all three cases, the size of the tip in contact was kept at 1.2 µm ensuring constant β = 1.2. As shown in Figure 7, the wedge angle has significant effect on pull-off stress for relatively high χ values where a crack initiates at the edge for both the rounded and 90° wedge. Below a critical χ*,* the pull-off stress for all three cases follow the same path once a crack is initiated at the center indicating little dependence on the wedge angle. Thus, a qualitative argument suggests that while fibers with small diameters and high elastic modulus (large χ) favor wedge-angle independence, the dependence of pull-off stress to the wedge angle is significant for high strength interfaces (small χ). This is assuming that the critical separation distance is in the order of 1 nm and somewhat constant for van der Waals interactions.

## Conclusion

In summary, the pull-off stress for an individual mushroom-like fiber was modelled using DB cohesive zone model and FE analyses. This study revealed critical information about the detachment mechanism of mushroom-like fibers and how this behaviour is influenced by the geometry as well as the interfacial properties. A simple geometrical guideline to ensure high and robust adhesion relative to the intrinsic adhesive stress was offered. While these results are important for designing dry fibrillar adhesives, they are only concerned with the performance when the loading is axial. The effect of shear loading should also be considered along with the results of this study in designing fibrillar adhesives.

## Supporting Information

File 1 Details of mathematical modeling.
