# Supplementary material for: The optimal shape of elastomer mushroom-like fibers for high and robust adhesion
Source: Beilstein J Nanotechnol. 2014 May 14;5:630–8. doi: 10.3762/bjnano.5.74 (PMC4077298; doi:10.3762/bjnano.5.74)
Supplement: File 1 — Details of mathematical modeling. [file Beilstein_J_Nanotechnol-05-630-s001.pdf]

Supporting Information

for

The optimal shape of elastomer mushroom-like fibers for high and robust adhesion

Burak Aksak*[1], Korhan Sahin[2] and Metin Sitti[3]

Address: [1]Department of Mechanical Engineering, Texas Tech University, Lubbock, TX 79409, USA; [2]Aerospace Engineering, University of Illinois at Urbana-Champaign, Urbana, IL 61801, USA and [3]Department of Mechanical Engineering, Carnegie Mellon University, Pittsburgh, PA 15213, USA

Email: Burak Aksak* - burak.aksak@ttu.edu

*Corresponding author

Details of mathematical modeling

Calculation of order of stress singularity

Bogy's study [1] on stress singularities at bimaterial wedge interfaces is used to determine the stress at the vicinity of the contact edge. Figure S1a is an illustration of a mushroom-like fiber and a smooth substrate.

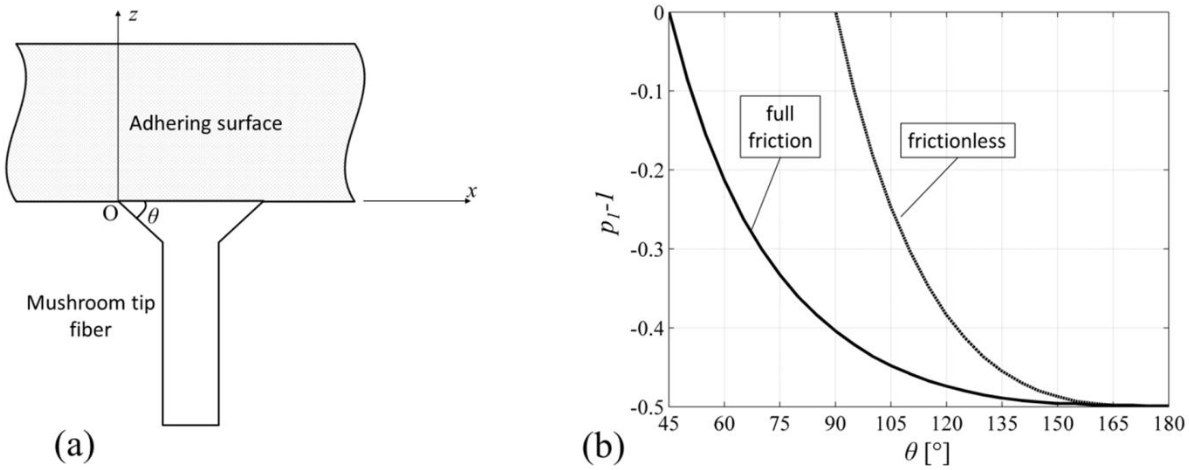

Figure S1: (a) An illustration of a mushroom-like fiber attached to a rigid smooth substrate under full friction condition. (b) Order of singularity ($p_1 - 1$) calculated for a full-friction interface by using Equation S1 and a frictionless interface by using Equation S2 at the tip apex as a function of the edge angle θ. Stresses are finite for θ ≤ 45° and singular for θ > 45° at the tip apex for a full-friction interface. The singular stress threshold increases to 90° for a frictionless contact. All of the analyses in this work are carried out for a full-friction interface.

Full-friction contact

Here, the fiber material is a soft incompressible elastomer (ν = 0.5) and the substrate material is rigid. For such bimaterial interfaces, the order of stress singularity can be calculated simply by finding the roots of

$$\mathcal{D} = \cos^2(p\theta) - p^2 \sin^2(\theta)\,. \tag{S1}$$

Let p_1 be real and the smallest root of $\mathcal{D}$ in $0 < p < 1$. If p_1 exists, then the stresses are singular at $d = 0$, where d is the distance from the edge of contact. The magnitude of the normal stress at the vicinity of the wedge apex is $\sigma_z = O(d^{-\alpha})$ where $\alpha = 1 - p_1$. If no zero of $\mathcal{D}$ in $0 < p < 1$ and $d\mathcal{D}/dp \neq 0$ when $p = 1$,

then the normal stress is finite, $\sigma_z = O(1)$. Figure S1b plots the smallest root of $\mathcal{D}$ as a function of the wedge angle θ. For θ > 45º, p_1 is real and the normal stress is singular at the contact edge. For θ ≤ 45º, there exists no root of $\mathcal{D}$ smaller than one and the normal stress at the contact edge is finite. Thus, edge angles less than 45º result in finite stresses at the interface.

Frictionless contact

Equation 1 is valid for perfectly bonded surfaces at which relative tangential displacement is constrained, i.e., full-friction interfaces. For slip boundaries at which there is no friction between the surfaces, and one of the adhering surfaces is much softer than the opposing surface; one can use the analysis provided by Gdoutos and Theocaris [2]. Assuming that the friction coefficient between the surfaces is zero, one can obtain the order of stress singularity by finding the roots of

$$\mathcal{D} = \sin(2p\theta) - p\sin(2\theta)\,. \qquad \text{(S2)}$$

Pull-off load calculation for array measurements

The load measured during an adhesion experiment is the total of the load contribution of each fiber in contact with an indenter. The relative displacement and the shape of the indenter dictate the deformation of each individual fiber in the array. This information can be used to calculate the overall load as a function of the relative displacement provided that the relation between deformation and load is known for an individual fiber. Assuming the fiber material is linear elastic, the relationship between axial load and deformation is defined by using a linear spring constant *k*. The maximum tensile load an individual fiber can exert on the indenter is limited by the pull-off load,

$$p_s = \pi a_t^{\,2}\sigma_s\,. \qquad \text{(S3)}$$

This implies that a fiber loses contact once the resultant load due to stretching on the fiber reaches p_s. The magnitude of deformation at the onset of pull-off can be calculated by using the linear spring constant k as

$$\Delta_s = \frac{p_s}{k} = \frac{\pi a_t^{\,2} \sigma_s}{k}. \qquad \text{(S4)}$$

As illustrated in Figure S2, for adhesion experiments performed on an array of fibers with a rigid backing using a hemispherical indenter of radius R, the highest tensile load (pull-off) is reached when the fibers contacting the tip of the hemisphere return to their nominal height in the retraction step [3,4]. At this instance, the outermost fibers are stretched by Δ_s since contact cannot be sustained beyond this deformation. By using this information, one can calculate the apparent contact radius c between the indenter and the fiber geometrically as

$$c = (2R\Delta_s)^{1/2}. \qquad \text{(S5)}$$

Assuming there are ρ number of fibers per unit area in the fiber array, the number of fibers in contact at pull-off can be calculated from the apparent circular contact area at pull-off,

$$N = \rho\pi c^2 = 2\rho\pi R\Delta_s. \qquad \text{(S6)}$$

The force an individual fiber exerts on the hemispherical indenter depends on the location where it contacts the indenter. Since the topography of the hemisphere is known and the fibers in contact should follow this topography, we have the deflection information, and thus know the load each fiber exerts on the indenter. Since load and deformation are linearly related to each other, one can define an average extension Δ_{av} for all fibers in contact at pull-off. This is done by finding the height h of a cylinder with radius c whose volume is equal to the portion of the hemisphere in contact with the array. This average extension is

$$\Delta_{av} = \Delta_s - h = \frac{\Delta_s}{3}\left(\frac{3R - 2\Delta_s}{2R - \Delta_s}\right) \approx \frac{1}{2}\Delta_s. \qquad \text{(S7)}$$

The approximation $\Delta_{av} \approx \Delta_s/2$ is valid only when $\Delta_s << R$. The pull-off load for the array of fibers can then be calculated as

$$P_s = k\sum_{i=1}^{N}\Delta_i = Nk\Delta_{av} \approx \frac{1}{2}Nk\Delta_s . \tag{S8}$$

Inserting Equation S4 and Equation S6 into Equation S8 yields

$$P_s = \frac{\rho R \pi^3}{k} a_t^{\ 4} \sigma_s^{\ 2} . \tag{S9}$$

Assuming the same indenter is used to test both the cylindrical and mushroom-like fiber arrays, ρ and k are the same for both arrays, and the base radius a of an individual mushroom-like fiber in the array is equal to the radius of a cylindrical fiber. The ratio of the mushroom-like fiber array pull-off force $P_{s,m}$ to the pull-off force obtained from the cylindrical fiber array $P_{s,c}$ becomes

$$e = \frac{P_{s,m}}{P_{s,c}} = \beta^4 \left(\frac{\sigma_{s,m}}{\sigma_{s,c}}\right)^2 . \tag{S10}$$

Here, subscripts m and c stand for mushroom-like and cylindrical fiber arrays, respectively.

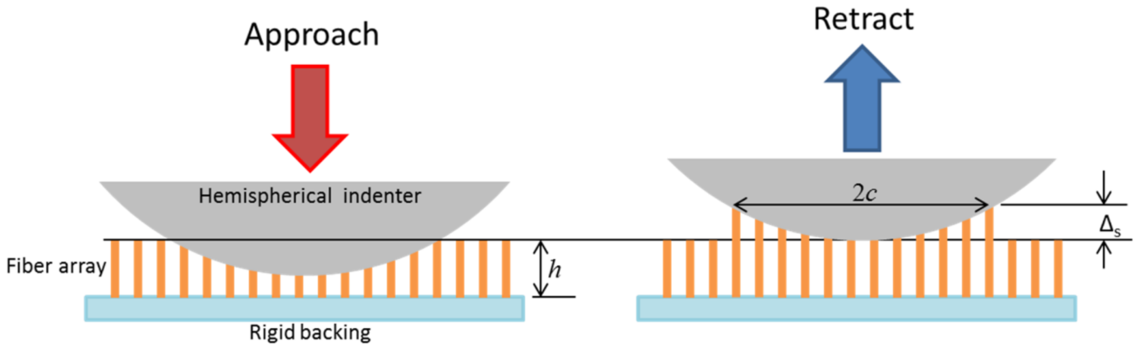

Figure S2: Schematics of a hemispherical indentation test to measure the pull-off load for an array of fibers. After the fibers are compressed with the indenter (approach), the indenter is retracted from the

fiber arrays. Maximum tensile load (pull-off load) is achieved when the fiber attached to the middle of the indenter is back to its nominal height *h* [3,4].

References

1. Bogy, D. B. *J. Appl. Mech.* **1971,** *38*, 377–386. doi:10.1115/1.3408786
2. Gdoutos, E. E.; Theocaris, P. S. *J. Appl. Mech.* **1975,** *42*, 688–692. doi:10.1115/1.3423663
3. Schargott, M.; Popov, V. L.; Gorb, S. *J. Theor. Biol.* **2006,** *243,* 48–53. doi:10.1016/j.jtbi.2006.05.023
4. Long, R.; Hui, C. Y. *Proc. R. Soc. A* **2009,** *465*, 961–981. doi:10.1098/rspa.2008.0362
